# Analysis of direct clinical consequences of MLC positional errors in volumetric‐modulated arc therapy using 3D dosimetry system

**DOI:** 10.1120/jacmp.v16i5.5515

**Published:** 2015-09-08

**Authors:** Karthikeyan Nithiyanantham, Ganesh K. Mani, Vikraman Subramani, Lutz Mueller, Karrthick K. Palaniappan, Tejinder Kataria

**Affiliations:** ^1^ Department of Radiation Oncology St. John's Medical College and Hospital Bangalore Karnataka India; ^2^ Department of Radiation Physics Kidwai Memorial Institute of Oncology Bangalore Karnataka India; ^3^ Division of Radiation Oncology Medanta Cancer Center, Medanta The Medicity Gurgaon Haryana India; ^4^ IBA Dosimetry Nurnberg Germany; ^5^ Research and Development Bharathiar University Coimbatore Tamilnadu India

**Keywords:** MLC positional accuracy, 3D dosimetry, VMAT

## Abstract

In advanced, intensity‐modulated external radiotherapy facility, the multileaf collimator has a decisive role in the beam modulation by creating multiple segments or dynamically varying field shapes to deliver a uniform dose distribution to the target with maximum sparing of normal tissues. The position of each MLC leaf has become more critical for intensity‐modulated delivery (step‐and‐shoot IMRT, dynamic IMRT, and VMAT) compared to 3D CRT, where it defines only field boundaries. We analyzed the impact of the MLC positional errors on the dose distribution for volumetric‐modulated arc therapy, using a 3D dosimetry system. A total of 15 VMAT cases, five each for brain, head and neck, and prostate cases, were retrospectively selected for the study. All the plans were generated in Monaco 3.0.0v TPS (Elekta Corporation, Atlanta, GA) and delivered using Elekta Synergy linear accelerator. Systematic errors of +1,+0.5,+0.3,0,−1,−0.5,−0.3 mm were introduced in the MLC bank of the linear accelerator and the impact on the dose distribution of VMAT delivery was measured using the COMPASS 3D dosimetry system. All the plans were created using single modulated arcs and the dose calculation was performed using a Monte Carlo algorithm in a grid size of 3 mm. The clinical endpoints $D_{95\%},D_{50\%},D_{2\%}$, and $D_{\text{max}},D_{20\%}$, D50% were taken for the evaluation of the target and critical organs doses, respectively. A significant dosimetric effect was found for many cases even with 0.5 mm of MLC positional errors. The average change of dose $D_{95\%}$ to PTV for ±1 mm,±0.5 mm, and ±0.3 mm was 5.15%, 2.58%, and 0.96% for brain cases; 7.19%, 3.67%, and 1.56% for head and neck cases; and 8.39%, 4.5%, and 1.86% for prostate cases, respectively. The average deviation of dose $D_{\text{max}}$ was 5.4%, 2.8%, and 0.83% for brainstem in brain cases; 8.2%, 4.4%, and 1.9% for spinal cord in H&N; and 10.8%, 6.2%, and 2.1% for rectum in prostate cases, respectively. The average changes in dose followed a linear relationship with the amount of MLC positional error, as can be expected. MLC positional errors beyond ±0.3 mm showed a significant influence on the intensity‐modulated dose distributions. It is, therefore, recommended to have a cautious MLC calibration procedure to sufficiently meet the accuracy in dose delivery.

PACS number: 87.56

## I. INTRODUCTION

The concept of intensity modulation in radiation therapy has an increased potential for the delivery of a homogeneous dose distribution to the tumor region while sparing the critical organs and normal structures to a greater extent. This has increased the feasibility of dose escalation for better tumor control probability and of dose reduction in order to lower normal tissue complication probability. The rotational dimension of intensity modulation was originally proposed by Yu^(1)^ in 1995 and later developed by Otto^(2)^ into volumetric‐modulated arc therapy (VMAT) in which the gantry angle and speed, multileaf collimator (MLC) leaves position, and dose rate varies simultaneously during radiation delivery. VMAT has a better delivery efficiency for highly conformal dose distribution compared to step‐and‐shoot or dynamic IMRT techniques. The increasing use of all these highly conformal techniques, which require all the deployment of MLCs, gives more and more importance to accurate function of this device. The MLCs are playing the major role in the modulation of beam by creating multiple segments to deliver the uniform dose distribution to the target with maximum sparing of normal tissues. For the accurate dose delivery, a stringent quality assurance program is required for the complex MLC system, as the intensity‐modulated beam delivery uses many small segments. Those are prone to have more variation in the output, even with minimum positional errors. Thus, the position of each MLC leaf has become extremely critical for the delivery of intensity‐modulated radiation beams. Moreover, unlike conventional beam (including 3D conformal) where only the peripheral region of dose distribution is affected, the intensity‐modulated delivery with MLC positional errors has a direct impact on the entire dose distribution. Many authors have discussed the evaluation of MLC positioning error and its impact on the fluence distribution for intensity‐modulated delivery technique, measuring with detector arrays and using 2D analysis with different passing criteria applied.^3^, ^4^, ^5^ Some authors have also utilized a manual editing of the MLC positions and applied a known magnitude of error. Subsequently, these configurations were reimported into the TPS in order to study the changes in dose distribution.^6^, ^7^ Most of the studies on MLC positional error — both random and systematic errors were analyzed — have reported that the random errors were insignificant, while systematic errors have shown significant effects on dose distributions, even with only 1 mm of positional errors applied for MLC positions.^3^, ^6^, ^7^ In this study, the MLC positional error impact on the dose distribution was analyzed using a patient‐specific 3D dose verification system (COMPASS; IBA Dosimetry, Schwarzenbruck, Germany). To our best knowledge, this is the first time that the direct clinical consequence of MLC positional errors in volumetric‐modulated arc therapy was analyzed using a 3D dosimetry system. The verification tool COMPASS uses a 2D ion chamber detector array (MatriXX) (IBA Dosimetry) for the measurement of IMRT and rotational plan delivery. Instead of using plastic phantoms (and hybrid dose distributions recalculated for these — usually homogeneous — phantoms), the real patient anatomy is used for the evaluation of a three‐dimensional dose distribution. Based on patient CT images and plan data, COMPASS precisely calculates patient doses rather than giving predictions, as is done by more rudimentary solutions that just superimpose CT images on dose estimates.^8^, ^9^, ^10^, ^11^ COMPASS uses a measurement‐based correction methodology, based on the prediction of the detector response in the MatriXX measurement plane prior to the measurement. This prediction is based on the read‐in collimator apertures (from DICOM RTplan), a commissioned beam model and the high resolution Monte Carlo‐derived detector response model (both spectral and spatial response). The dose determination in COMPASS combines a fluence prediction based on plan input, and commissioned machine model with a perturbational correction based on the discrepancy between measured and predicted responses. As the fluence calculation, on the one hand, is done in a 2 mm grid, the native resolution of the array detector, on the other hand, is 7.6 mm, this perturbational approach helps achieve maximum resolution for the reconstructed dose. For each delivered segment (defined as the interval between two control points), the response difference is split in a global linear (rescaling of the segment fluence) and a local ‘residual’ term. While the first term can be directly applied to rescale the delivered fluence (in the 2 mm resolution), the second term gives, after a deconvolution, the detector native resolution perturbational correction. The accuracy of this algorithm has been evaluated in a paper by Godart et al.,^(9)^ where it has been benchmarked against a film measurement. Based on these results, the impacts of MLC positional error on the dose distribution was measured for intentionally introduced systematic errors of +1,+0.5,+0.3,−1,−0.5, and −0.3 mm for the MLC leaf banks.

## II. MATERIALS AND METHODS

### A. Patient selection and treatment planning

A total of 15 VMAT patient plans, five each for brain, head and neck and prostate cases, respectively, were selected for a retrospective analysis in this study. The brain cases consisted in one brainstem glioma, one anaplastic glioma, one astrocytoma, and two cases of glioblastoma (GBM). For the H&N sites, one case of base of tongue, hard palate, oropharynx, larynx, and postcricoid were chosen. For prostate cases, three out of the five patients had a regional node involvement.

The treatment goal for the brain cases was to deliver doses between 54–66 Gy to 95% of the planning target volume (PTV), and for H&N cases, 54Gy–70Gy to 95% of the PTV, while simultaneously meeting the plan acceptance criteria for critical structures. For the prostate cases without regional nodal involvement, a dose of 72 Gy was selected; for those with nodal involvement, a phase 1 simultaneous integrated boost (SIB) plan with dose of 45 Gy to nodes and of 50 Gy to planning gross tumor volume. A coverage of 95% was set as a goal in these cases. The dose per fraction for all cases was 1.8 or 2 Gy. These 15 plans comprised both simple and complex plans in terms of the involvement of critical organs in the PTV and its involvement of critical organs, especially when close to PTV. All plans were generated in Monaco v3.0.0 treatment planning system (TPS) (Elekta Corporation, Atlanta, GA) using Monte Carlo dose calculation algorithm with 6 MV photon beams. The treatment planning in Monaco is a two‐step process that calculates the optimal fluence in the first step and converts it into deliverable MLC segments in the later step.^12^, ^13^, ^14^ The treatment plan parameters, such as arc length, arc increment, arc start/stop angle, and minimum segment width, were handled based on tumor type and site. A calculation grid of 3 mm was used for all the plans.

### B. Linear accelerator and MLC positional error simulation

All the plans were delivered using Elekta Synergy linear accelerator (Elekta Ltd., Crawley, UK) equipped with the ‘Beam Modulator’ head which consists of 40 pairs of leaves each 4 mm wide (projected to isocenter). This MLC has the capability of interdigitation. The MLC controller uses an optical system for the determination of leaf positions. A reflector is attached at each leaf end and illuminated with a light source. The leaf position is observed via a mirror assembly and a charged coupled device (CCD) camera‐based imaging device which is interfaced to a control computer. The optically read‐out position and the leaf position determined are connected via a linear calibration using an offset and a gain parameter. Offset and gain is defined as ‘major’ and ‘minor’ values, where the major offset/gain acts on the whole MLC, the minor value on an individual leaf. Using the digital controller for the linear accelerator RT Desktop 7.01 (Elekta Ltd.), the leaf positions were adjusted by changing the leaf offset values in order to introduce systematic leaf position errors. The MLC controller of the beam modulator head assembly was set as default to 26 units of leaf offset (equivalent to 1 mm of leaf movement at isocenter). Based on these adjustments, systematic errors of +1,+0.5,+0.3,−1,−0.5, and −0.3 mm were introduced in both the leaf banks. The resultant dose distributions were compared with the original plan generated with 0 mm shift of the leaf banks.

### C. COMPASS 3D dosimetry

COMPASS is a system for clinically relevant 3D treatment verification and patient dose analysis. COMPASS reconstructs dose from measured fluence, compares the patient plan with measurements, and provides 3D dose deposition information inside the patient's anatomy. Plan evaluation is achieved either by visual means (evaluating dose differences/gamma relative to TPS inside patient CT) or on a structure‐by‐structure, statistical/quantitative basis via comparison of the TPS generated DVHs to that of COMPASS's independently determined DVHs. Using an individually commissioned beam model, the COMPASS system calculates 3D dose distributions, as in a TPS. The DICOM files RT plan, RT dose, RT structures, and CT images are imported from the TPS. The COMPASS uses the collapsed cone convolution algorithm^(15)^ for dose calculation based on patient and plan data with the help of the commissioned individual beam model. Data evaluation is performed in 3D, including a dose‐volume histogram (DVH) comparison. In addition, the COMPASS system recomputes the dose delivered based upon the measurement with the 2D array detector; by predicting the detector response by means of a hard‐coded Monte Carlo detector model (both spatial and spectral response function) for ‘perfect’ delivery and comparing this value with the measured response, a perturbational correction is applied to the ‘ideal’ fluence in order to get the actual delivered one. This ‘measured’ fluence is then used for the 3D dose comparison. The treatment plans were delivered on MatriXX with the following measurement setup: 2 cm water‐equivalent buildup and 100 cm source‐to‐detector plane distance (SDD) by mounting it on the gantry using a holder. The MatriXX was connected to a gravity‐based inclinometer fixed at the gantry. Thus the COMPASS system acquires the delivered fluence and simultaneously the gantry angle for the dose reconstruction on patient anatomy given by the CT dataset.

### D. Data analysis

The 3D dose distributions reconstructed on patient data in COMPASS software were analyzed for all MLC positional error conditions. The dose distributions reconstructed with 0 mm shift of the MLC leaf banks were taken as a reference to which all other plans were compared. For quantitative analysis, the endpoints for dose $D_{95\%},D_{50\%}$, and $D_{2\%}$ were selected for planning target volumes for all the cases. As critical organs for H&N cases, dose $D_{\text{max}}$ to brainstem and spinal cord and the dose $D_{50\%}$ to parotids, paranasal sinus (PNS) oral cavity, mandible, temporomandibular (TM) joints, cochleae, larynx, and trachea were analyzed. For brain cases, dose $D_{\text{max}}$ to brainstem, optic chiasm, optic nerves and dose $D_{20\%}$ for eyes and $D_{50\%}$ for cochleae were analyzed, respectively. For prostate cases, dose $D_{20\%}$ to rectum and bladder, $D_{50\%}$ to femurs and pelvic bones, and $D_{\text{max}}$ to bowel were taken for the analysis.

## III. RESULTS

COMPASS, the 3D dosimetry tool was used to measure the VMAT plans with intentionally introduced MLC errors for three different tumor sites, and the results are shown in 1, 2, 3. The negative and positive values of the errors in the MLC position offset, which in fact shrink or enlarge the radiation portals, result in an observed overall reduction or increase of the doses.

**Figure 1:**
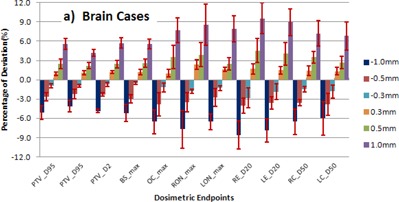
The mean and error bars of SD at different endpoint doses of targets and critical organs at risk for brain cases and MLC positional errors of −1,−0.5,−0.3,+0.3,+0.5, and +1 mm with respect to original plan (0 mm). PTV = planning target volume, BS = brainstem, OC = optic chiasm, RON = right optic nerve, LON = left optic nerve, RE = right eye, LE = left eye, RC = right cochlea, LC = left cochlea.

**Figure 2:**
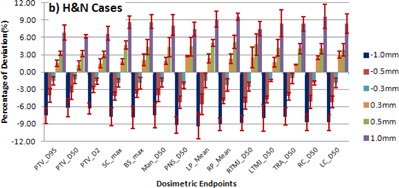
Mean values and error bars of standard deviation for different endpoint doses of targets and critical organs for head and neck cases. MLC positional errors of −1,−0.5,−0.3,+0.3,+0.5, and +1 mm with respect to original plan where introduced. PTV = planning target volume, SC = spinal cord, BS = brainstem, Man = mandible, PNS = paranasal sinuses and oral cavity, LP = left parotid, RP = right parotid, RTMJ = right temporomandibular joint, LTMJ = left temporomandibular joint, TRA = trachea and larynx, RC = right cochlea, LC = left cochlea.

**Figure 3:**
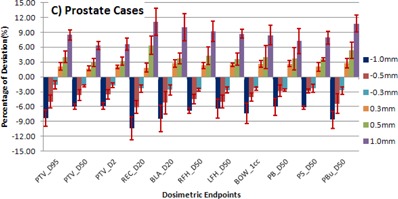
The mean and SD for dosimetric endpoints of targets and critical organs for prostate cases and MLC positional errors of −1,−0.5,−0.3,+0.3,+0.5, and +1 mm with respect to original plan (0 mm). PTV = planning target volume, REC = rectum, BLA = bladder, RFH = right femur head, LFH = left femur head, BOW = bowel, PB = pelvic bones, PS = penis and scrotum, PBu = penile bulb.

### A. Brain cases

**Figure 4:**
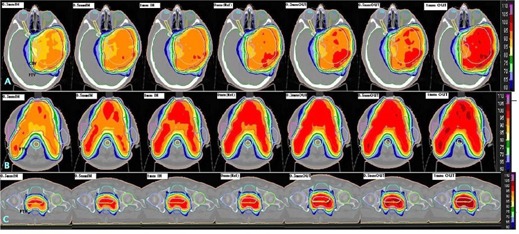
The dose distribution calculated by COMPASS 3D dosimetry system showing the dose variations caused by MLC positional error. The panels A, B, and C represent the brain, head and neck and prostate cases. In each panel the central image shows the reference dose distribution (0 mm error) and images from the left to right correspond to MLC positional errors of −1,−0.5,−0.3,0,+0.3,+0.5, and +1 mm, respectively.

For the brain cases, the mean and standard deviation (SD) of dose $D_{95\%},D_{50\%}$, and $D_{2\%}$ of planning target volume (PTV), dose $D_{\text{max}}$ for brainstem, optic chiasm, and optic nerves, dose $D_{20\%}$ for eyes, and dose $D_{50\%}$ for cochleae are shown in Fig. 1. Among these critical organs, some were inside or partially covered by the PTV, some were proximally situated, and others were far away. The range of dose $D_{95\%},D_{50\%}$, and $D_{2\%}$ for PTV measured with MLC positional errors of −1mm to +1 mm for these five patients have a mean ± SD of ‐5.15%±1.07% to 5.57%±0.88%,‐4.11%±0.92% to 4.15%±0.53%, and ‐3.87%±0.24% to 5.71%±0.82%, respectively. The maximum difference was observed with an error of −1mm and results in a reduction of target coverage ($D_{95\%}$) by 6.27%. In our measurements for the five brain cases, the extreme positive error of +1 mm in MLC position results in a wider radiation portal showing an increased mean ± SD value for $D_{\text{max}}$ of brain stem, optic chiasm, right optic nerve, and left optic nerve as 5.56%±0.7%,8.20%±2.55%,8.60%±3.20%, and 7.92%±1.99%, respectively. For the other extreme (i.e., −1mm of MLC error) reduces the radiation portals and decreases the dose $D_{\text{max}}$ to a mean ± SD of ‐5.18%±1.33%,−6.55%±1.85%,−7.70%±2.90%, and −5.54%±1.21%, respectively. The average changes of dose $D_{50\%}$ to right and left eye for the range of −1mm to +1 mm of MLC positional errors were observed as −7.59%±2.15% to 9.56%±2.40% and −7.26%±1.81% to −8.97%±0.8%. For the same range of MLC errors, the changes in dose $D_{50\%}$ of right and left cochlea were −6.54%±1.14% to 7.22%±0.94% and −6.00%±2.62% to 6.82%±2.17%. Figure 4 demonstrates the dose distribution changes after introducing the systematic errors of −1 mm,−0.5 mm,−0.3 mm,+0.3 mm,+0.5 mm, and +1 mm in MLC positional calibration. The negative systematic errors, which reduce each segment size, result in underdose; the target coverage vs. introduced error is shown in Fig. 1. On the other hand, positive systematic errors enlarge each segment and subsequently result in overdose to the target and the creation of new hot spots inside the target volume, as well as in an increase of the dose to critical organs at risk.

### B. Head and neck cases

For head and neck cases, Fig. 2 shows the dosimetric variation due to the MLC positional error. Mean ± SD values of dose variation using endpoints like $D_{95\%},D_{50\%},D_{2\%}$ for PTV and $D_{\text{max}}$ for spinal cord and brainstem, and $D_{50\%}$ for other critical organs like mandible, PNS oral cavity, right/left TM joint, trachea, and right/left cochlea were determined. For right and left parotid, the mean dose was chosen as an endpoint. The statistical results of the mean ± SD of dose variation due to the MLC positional error of −1mm to +1 mm for $D_{95\%},D_{50\%}$, and $D_{2\%}$ of target volume were ‐7.56%±1.34% to 6.82%±1.28%,‐6.22%±1.53% to 6.17%±0.34%, and ‐6.34%±0.99% to 6.67%±1.19%, respectively. Due to shrinkage and expansion of radiation portals by 1 mm on both the leaf banks, the maximum difference in target coverage $D_{95\%}$ was observed as −8.78% and 8.65%, respectively. Similar to the situation for serial organs in the brain cases, the simulation of MLC error of +1 mm has increased the dose $D_{\text{max}}$ for spinal cord and brainstem by 10.1% and 9.82%, respectively. The range of mean ± SD changes of right and left parotid's endpoint dose were observed as ‐9.08%±1.03% to 9.60%±0.57% and −9.36%±2.26% to 9.14%±1.35%, respectively. For other critical organs like mandible, PNS oral cavity, right TM joint, left TM joint, right cochlea, and left cochlea, the range of mean ± SD changes of dose $D_{50\%}$ resulted in −7.49%±2.16% to 8.11%±1.79%,−9.21%±1.47% to 7.45%±1.19%,−8.88%±1.33% to 7.47%±1.22%,−7.96%±2.31% to 8.44%±2.33%,−8.68%±1.44% to 9.65%±2.07%, and −8.74%±1.36% to 8.37%±0.70%, respectively.

### C. Prostate cases

Figure 3 demonstrates the difference in dose $D_{95\%},D_{50\%}$, and $D_{2\%}$ for PTV and appropriate endpoints for critical organs like rectum, bladder, right/left femoral head, pelvic bones, penis scrotum, and penile bulb for different MLC positional errors in the five prostate cases. For PTV, a range of mean ± SD changes from ‐7.56%±1.34% to 6.82%±1.28%,‐6.22%±1.53% to 6.17%±0.34%, and ‐6.34%±0.99% to 6.67%±1.19% was observed at dose $D_{95\%},D_{50\%}$, and $D_{2\%}$, respectively, for MLC positional errors from −1mm to +1 mm. For rectum and bladder as critical organs, the $D_{20\%}$ was used as the endpoint, and the range of changes resulted in ‐10.39%±2.31% to 11.11%±2.69% and −8.42%±2.68% to 10.02%±2.69%, respectively. The ROIs right femoral head, left femoral head, pelvic bones, penis scrotum, penile bulb ($D_{50\%}$), and bowel ($D_{\text{max}}$) showed a mean ± SD of −6.82%±1.57% to 9.21%±2.03%,−6.40%±1.95% to 8.70%±0.88%,−6.04%±1.71% to 7.25%±2.54%,−6.19%±0.39% to 7.90%±1.36%,−8.62%±1.71% to 10.76%±1.72%, and −7.39%±2.04% to 8.39%±1.98%, respectively.

## IV. DISCUSSION

Performing an MLC calibration procedure is mandatory under many circumstances, such as the commissioning of TPS, commissioning of delivery system, mechanical alterations in the MLC control system, and the periodical QC procedure.

Many studies have revealed that even 1 mm of error in MLC position can produce significant changes in intensity‐modulated dose distributions because of the presence of numerous narrow apertures in highly modulated radiation therapy. More stringent analysis of the fluence with MLC positional error using 2% of dose difference and 1 mm distance to agreement can help to identify the accurate positioning of MLC.^16^, ^17^, ^18^, ^19^ Mu et al.^(20)^ reported that a systematic MLC positional error of 1 mm could change the target dose $D_{95\%}$ by 4% for a simple plan and 8% for a more complex plan. The impact of 1 to 2 mm of MLC positional errors was studied indirectly by several authors, who have reported 5% to 8% change in $D_{95\%}$ for target coverage, and up to 12% in dose $D_{0.01\%}$ to critical organs.^5^, ^6^ Also, the surrogate of editing MLC DICOM files and recalculating in TPS has shown that the severe MLC positional error already begins in the range of 0.3 to 0.6 mm of deviation, as the target dose discrepancy exceeds 2%.^4^, ^6^, ^7^ However, 3D dosimetry is the appropriate choice to study the dose volume effects on target and critical organs at actual scenario. This allows the evaluation of MLC positional error consistency under more realistic delivery of the intensity‐modulated arc beam geometries that includes mechanical backlash due to gravity and gantry sag.

In the results of our direct measurement using 3D dosimetry, we observed that for head and neck and prostate cases, the average deviation of target coverage $D_{95\%}$ was 2.41% to 3.16% higher compared to brain cases, due to the increased degree of intensity modulation for those plans. From 1, 2, 3 we observed for the selected endpoints that the dose to critical organs at risk for the different cases shows a significant deviation for any of the given MLC positional errors. The results for the various endpoints and patients treated at the same sites can significantly vary. This depends on the location of the organ at risk relative to the target structure (distal‐proximal‐partially covered) and on the size of the ROI structure. The highest deviations were obviously found for ROIs partially within the target structure and for very small ROIs. The average change in dose to PTV and the critical organs at risk approximately follows the predicted linear relationship with a negative and positive magnitude of MLC positional errors. The average change of dose $D_{95\%}$ to PTV for ±1 mm,±0.5 mm, and ±0.3 mm were 5.15%, 2.58%, and 0.96% for brain cases, 7.19%, 3.67%, and 1.56% for head and neck cases, and 8.39%, 4.5%, and 1.86% for prostate cases, respectively.

Table 1 summarizes the variation of dose distribution due to MLC positional error in the VMAT plan for both target and critical organs at risk for all the cases. In most of the circumstances the critical organs like brain stem, optic chiasm, parotids, mandible, rectum, and bladder are partially involved into the target volume and, therefore, demand very steep dose gradients to spare the normal structure as much as possible. For example, in the cases involving brain stem, introducing an MLC positional error of ±1 mm,±0.5 mm, and ±0.3 mm resulted in a maximum average dose deviation for $D_{\text{max}}$ of 5.4%, 2.8%, and 0.83%, respectively. On the other hand, the values for spinal cord and rectum resulted in 8.2%, 4.4%, 1.9%, and 10.8%, 6.2%, 2.1%, respectively. The results of selected endpoints for the parallel organs at risk followed the same trend. Likewise, the dose volume effect for smaller organs like optic chiasm, optic nerve, cochleae, and penile bulb shows the obvious differences for dose deviation with MLC positional error for the different cases.

Thus, accurate MLC calibration and leaf gap consistency are critical for the accurate delivery of dynamic intensity modulated beams. The verification of leaf positions and gap consistency has been reported by several authors. LoSasso et al.^(21)^ examined the accuracy and reproducibility of the leaf gaps by ion chamber measurements with sliding slit field dynamic MLC delivery. Chang et al.^(22)^ and Vieira et al.^(23)^ verified leaf gap consistency by checking the full width at half maximum (FWHM) of sliding slit beams at stopping positions measured, using electronic portal imaging device (EPID). Mei et al.^(24)^ has reported that the ion chamber measurements of the dosimetric leaf gap (DLG) can effectively check systematic MLC gap change of 0.2 mm and 2D detector can effectively check MLC gap consistency and detect changes in isolated areas away from central axis.

Our study results show that MLC positional errors in the range from 0.3 to 1.0 mm — in both positive and negative directions — will disturb the dose distribution in a linear relationship with respect to the magnitude of the error. From 1, 2, 3, 4 it is evident that the head and neck and prostate plans were slightly more sensitive to MLC positional errors than brain cases, as the former plans were prone to a higher degree of modulation. An MLC error of ±0.5 mm was resulting in a dose deviation of more than 3% when the plan demands for a high degree of modulation and/or a steep dose gradient. It turned out that the results were consistent with other studies on MLC positional error. This underlines the demand for a stringent quality assurance protocol to ensure the dose deviation lies within clinically acceptable tolerance.

**Table 1:** The percentage deviation of mean ± SD in the dosimetry endpoints calculated for brain, head and neck and prostate cases with systematic MLC positional error of ±0.3,±0.5, and ±1 mm with reference to original plan (0 mm error)

	*MLC Positional Error (mm)*												
	*End*	−1.0		−0.5		−0.3		*0.3*		*0.5*		*1.0*	
*Structure*	*Points*	*Mean*	*SD*	*Mean*	*SD*	*Mean*	*SD*	*Mean*	*SD*	*Mean*	*SD*	*Mean*	*SD*
*Brain Cases*													
PTV	$D_{95}$	−5.15	1.07	−2.67	0.65	−0.98	0.34	0.94	0.26	2.49	0.73	5.57	0.88
PTV	$D_{50}$	−4.11	0.92	−2.25	0.77	−0.96	0.21	1.10	0.34	2.19	0.52	4.15	0.53
PTV	$D_{2}$	−4.87	0.24	−2.29	0.27	−0.72	0.29	1.15	0.20	2.43	0.63	5.71	0.82
Brainstem	Max	−5.18	1.33	−2.94	0.62	−0.48	0.20	1.18	0.44	2.59	0.64	5.56	0.70
OC	Max	−6.55	1.85	−3.83	1.77	−1.20	0.72	1.03	0.60	3.55	1.80	7.70	1.88
RtOpticNerve	Max	−7.70	2.93	−3.52	1.48	−1.79	0.36	2.37	0.79	3.90	1.86	8.60	3.20
LtOpticNerve	Max	−6.54	1.21	−2.77	1.40	−1.31	0.36	1.66	0.33	2.51	0.97	7.92	1.99
Rt Eye	$D_{20}$	−8.59	2.15	−4.07	1.19	−2.83	1.57	1.70	0.82	4.54	1.84	9.56	2.40
Lt Eye	$D_{20}$	−7.86	1.81	−3.64	0.97	−1.89	1.22	1.47	0.58	4.02	1.79	8.97	2.08
Rt Cochlea	$D_{50}$	−6.54	1.94	−3.57	0.51	−1.46	0.43	1.38	0.78	3.49	0.94	7.22	1.94
Lt Cochlea	$D_{50}$	−6.00	2.62	−3.83	1.71	−1.84	1.01	1.34	0.58	2.70	0.97	6.82	2.17
*Head and Neck Cases*													
PTV	$D_{95}$	−7.56	1.34	−4.07	1.17	−1.57	0.69	1.55	0.52	3.26	0.39	6.82	1.28
PTV	$D_{50}$	−6.22	1.53	−3.61	1.15	−1.30	0.67	1.19	0.79	3.19	0.50	6.17	0.34
PTV	$D_{2}$	−6.34	0.99	−3.01	0.53	−1.62	0.56	1.48	0.80	3.09	0.54	6.67	1.19
Spinalcord	Max	−7.75	1.53	−4.14	0.73	−1.92	0.75	1.78	0.49	4.67	0.77	8.64	1.03
Brainstem	Max	−7.89	1.90	−3.76	0.62	−1.93	1.03	2.10	0.97	4.29	1.34	8.70	1.21
Mandible	$D_{50}$	−7.49	2.16	−4.08	1.07	−1.83	0.73	1.95	0.58	4.35	1.67	8.11	1.79
PNS	$D_{50}$	−9.21	1.47	−5.21	1.08	−2.29	0.53	2.75	0.16	4.40	1.60	7.45	1.19
Lt Parotid	Mean	−9.39	2.26	−5.61	1.78	−1.50	1.29	2.35	0.78	5.04	0.62	9.14	1.35
Rt Parotid	Mean	−9.08	1.03	−5.00	0.50	−2.27	1.06	2.29	0.87	5.19	1.17	9.60	0.57
Rt TMJ	$D_{50}$	−8.88	1.33	−5.38	1.02	−2.57	0.76	2.49	1.78	4.77	1.83	7.47	1.22
Lt TMJ	$D_{50}$	−7.96	2.31	−4.76	0.55	−1.46	0.18	1.71	0.83	4.19	1.44	8.44	2.33
Trachea	$D_{50}$	−7.73	1.49	−4.21	0.65	−1.12	0.92	1.29	0.09	4.02	0.85	8.36	1.23
Rt Cochlea	$D_{50}$	−8.68	1.44	−5.17	1.15	−1.94	0.37	2.48	0.28	4.03	0.88	9.65	2.07
Lt Cochlea	$D_{50}$	−8.74	1.36	−5.20	1.06	−1.88	0.80	3.03	0.60	3.84	1.12	8.37	1.70
*Prostate Cases*													
PTV	$D_{95}$	−8.31	1.59	−4.98	1.28	−1.63	0.81	2.09	0.75	4.02	1.17	8.47	1.05
PTV	$D_{50}$	−5.95	0.53	−3.62	1.10	−1.81	0.20	1.72	0.47	2.87	0.80	6.32	0.76
PTV	$D_{2}$	−5.92	0.68	−3.58	0.94	−1.69	0.47	2.03	0.32	3.19	0.78	6.61	1.20
Rectum	$D_{20}$	−10.4	2.31	−6.07	1.26	−2.36	0.71	1.86	0.86	6.37	1.80	11.11	2.69
Bladder	$D_{20}$	−8.42	2.68	−5.23	2.25	−2.64	1.02	2.72	0.73	3.71	1.13	10.02	2.69
Rt Fem Head	$D_{50}$	−6.82	0.57	−4.47	0.97	−2.61	0.26	2.33	0.62	4.25	1.75	9.21	2.03
Lt Fem Head	$D_{50}$	−6.40	1.95	−5.00	1.34	−2.67	0.66	2.44	0.38	3.62	1.21	8.70	0.88
Bowel	Max	−7.39	2.04	−4.08	0.85	−2.35	0.37	2.63	0.67	3.98	2.37	8.39	1.98
Pelvic Bones	$D_{50}$	−6.04	1.71	−2.83	1.14	−2.64	0.17	2.65	0.60	3.65	2.21	7.25	2.54
Penis/Scrotum	$D_{50}$	−6.19	0.39	−2.83	0.45	−2.31	0.85	2.05	0.89	3.51	0.31	7.90	1.36
Penile Bulb	$D_{50}$	−8.62	1.71	−5.40	1.93	−2.78	0.77	2.72	0.95	5.35	1.62	10.76	1.72

All the data are represented as percentage.

## V. CONCLUSIONS

We studied the impact of MLC positional errors using a 3D dosimetry system by intentionally introducing systematic errors in MLC leaf bank calibration. Using the COMPASS 3D dosimetric system, the consequences of the MLC positional error on different endpoint doses have shown the importance of accurate MLC calibration for intensity‐modulated arc therapy. Especially it has been proven that MLC positional error beyond ±0.3 mm can have a clinically relevant influence on the dose distribution; therefore, an MLC calibration procedure which can guarantee a precision close to this value is mandatory to meet the required accuracy in dose delivery. The importance of the MLC positioning accuracy has become more critical for the advanced treatment delivery techniques like VMAT and IMRT, which can produce very steep dose gradients using narrow collimator openings and therefore largely depend on accurate segment boundaries.

## Supporting information

Supplementary Material
