# Supplementary figures and images for: Analysis of direct clinical consequences of MLC positional errors in volumetric‐modulated arc therapy using 3D dosimetry system
Source: J Appl Clin Med Phys. 2015 Sep 8;16(5):296–305. doi: 10.1120/jacmp.v16i5.5515 (PMC5690184; doi:10.1120/jacmp.v16i5.5515)

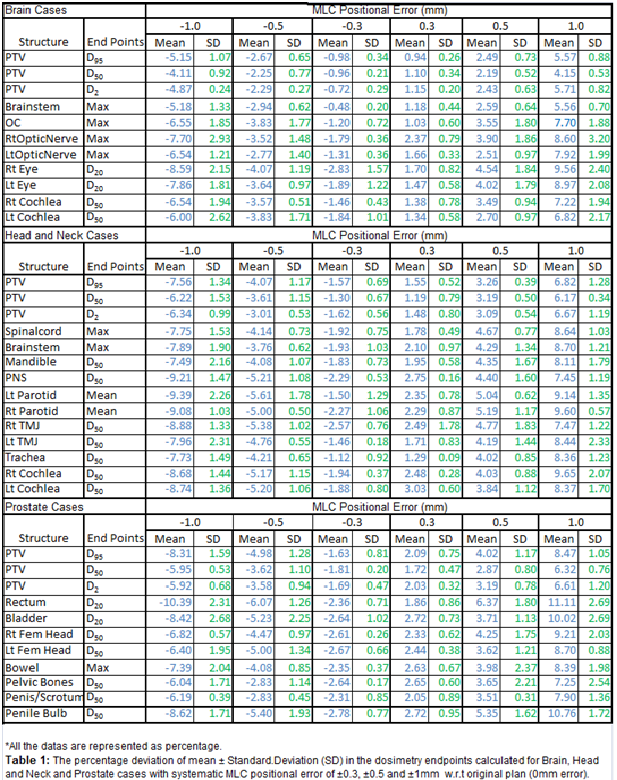

Supplement: Supplementary file 1 — Supplementary Material [file ACM2-16-296-s001.png]
